# What’s the Risk: Differentiating Risk Ratios, Odds Ratios, and Hazard Ratios?

**DOI:** 10.7759/cureus.10047

**Published:** 2020-08-26

**Authors:** Andrew George, Thor S Stead, Latha Ganti

**Affiliations:** 1 Emergency Medicine, Brown University, Providence, USA; 2 Emergency Medicine, Warren Alpert Medical School, Providence, USA; 3 Emergency Medicine, University of Central Florida College of Medicine, Orlando, USA; 4 Emergency Medicine, Envision Physician Services, Nashville, USA; 5 Emergency Medical Services, Polk County Fire Rescue, Bartow, USA

**Keywords:** hazard ratio, risk ratio, odds ratio

## Abstract

Risk ratios, odds ratios, and hazard ratios are three common, but often misused, statistical measures in clinical research. In this paper, the authors dissect what each of these terms define, and provide examples from the medical literature to illustrate each of these statistical measures. Finally, the correct and incorrect methods to use these measures are summarized.

## Introduction and background

Risk ratios, odds ratios, and hazard ratios are three ubiquitous statistical measures in clinical research, yet are often misused or misunderstood in their interpretation of a study’s results [1]. A 2001 paper looking at the use of odds ratios in obstetrics and gynecology research reported 26% of studies (N = 151) misinterpreted odds ratios as risk ratios [2], while a 2012 paper found similar results within published literature on obesity, with 23.2% of studies (N = 62) published across a one-year period in two leading journals misrepresenting odds ratios [3].

Understanding the three measures’ applicability and usage allows for a more accurate interpretation of study results and a better understanding of what each value demonstrates and, equally importantly, what each does not.

## Review

Confidence intervals and p-values

In order to entertain any discussion of statistical analysis, it is important to first understand the concept of population statistics. Plainly, population statistics are the values of any measure within the population of interest, and estimating them is the goal of most studies [4]. For instance, in a study looking at obesity rates for patients on a certain medication, the population statistic could be the average obesity rate for all patients on the medication.

However, identifying this value would require having data for every single individual that falls into this category, which is impractical. Instead, a randomized sample can be gathered, from which sample statistics can be obtained. These sample statistics serve as estimates of the corresponding population statistics and allow a researcher to make conclusions about a population of interest.

A significant limitation exists in that these constructed samples must be representative of the larger population of interest. While there are many steps that can be taken to reduce this limitation, sometimes its effects (so-called sampling bias [5]) go beyond the control of the researcher. Additionally, even in a theoretical situation with no sampling bias, randomization could result in a misrepresentative sample. In the previous example, suppose that the population rate of obesity among all adults eligible for the medication was 25%. In a simple random sample of 30 patients from this population, there is a 19.7% chance that at least 10 patients will be obese, resulting in a sample obesity rate of 33.3% or even higher. Even if there is no relationship between the medication and obesity rates, it is still possible to encounter a rate that appears to be different from the overall obesity rate, which occurred through randomness in sampling alone. This effect is the reason for reporting confidence intervals and p-values in clinical research.

Confidence intervals are intervals in which the population statistic could lie. They are constructed based on the sample statistic and certain features of the sample that gauge how likely it is to be representative and are reported to a certain threshold [6]. A 95% confidence interval is an interval constructed such that, on average, 95% of random samples would contain the true population statistic within their 95% confidence interval. Thus, a threshold for significant results is often taken as 95%, with the understanding that all values within the reported range are equally valid as the possible population statistic.

The p-value reports similar information in a different way. Rather than constructing an interval around a sample statistic, a p-value reports the probability that the sample statistic was produced from random sampling of a population, given a set of assumptions about the population, referred to as the “null hypothesis” [7]. Taking the example study on obesity rates again, the obesity rate among the sample (a sample of patients on the medication) could be reported alongside a p-value determining the chance that such a rate could be produced from randomly sampling the overall population of patients eligible for the medication. In the case of the study, the null hypothesis is that the population rate of obesity among patients on the medication is equal to the overall rate of obesity among all patients eligible for the medication, that is, 25%. A one-tailed p-value can be used if there is reason to believe that an effect would occur in only one direction (for instance, there may be reason to believe the medication would increase weight gain but not decrease it), whereas a two-tailed p-value should be used in all other cases. When using a symmetric distribution, such as the normal distribution, two-tailed p-values are simply twice the one-tailed p-value.

Suppose again that a sample of 30 patients on the medication contains 12 obese individuals. With a one-tailed test, our p-value is 0.0216 (using the binomial distribution). Thus, we can say that our observed rate of 40% is significantly different from the hypothesized rate of 25% at a significance level of 0.05. In another sense, the 95% confidence interval for the observed proportion is 25.6% to 61.07%. Confidence intervals correspond to two-tailed tests, where a two-tailed test is rejected if and only if the confidence interval does not contain the value associated with the null hypothesis (in this case, 25%).

If a calculated p-value is small, it is likely the population is not structured as originally stated in the null hypothesis. If we obtain a low p-value, we have evidence that there was some effect or reason for the observed difference - the medication, in this case. A threshold of 0.05 (or 5%) is typically used, with a p-value having to be below this threshold for its corresponding attribute to be statistically significant.

Risk ratios

Risk, another term for probability, is another fundamental principle of statistical analysis. Probability is a comparison of observing a specific event occurring as a result to the total unique results. A coin flip is a trivial example: the risk of observing a heads is ½ or 50%, as of all possible unique trials (a flip resulting in heads or a flip resulting in tails), only one is the event of interest (heads).

Using only risk allows predictions about a single population. For instance, looking at obesity rates within the U.S. population, the CDC reported that 42.4% of adults were obese in 2017-2018. So, the risk of an individual in the U.S. being obese is around 42.4% [8]. However, most studies look at the effect of a specific intervention or other item (such as mortality) on another. Earlier, we supposed that the obesity rate of eligible patients was 25%, but here we will use the 42.4% associated with the U.S. adult population. Suppose we observe a risk of 25% in a random sample of patients on the medication as well. To conceptualize the effect of the medication on obesity, a logical next step would be dividing the risk of obesity in the U.S. population on the medication with the risk of obesity in the U.S. population, which results in a risk ratio of 0.590.

This calculation - a ratio of two risks - is what is meant by the eponymous risk ratio (RR) statistic, also known as relative risk. It allows a specific number to be given for how much more risk an individual in one category bears compared to an individual in another category. In the example, an individual taking the medication bears 0.59 times as much risk as an adult from the general U.S. population. However, we have assumed that the population eligible for the medication had an obesity rate of 25% - perhaps only a group of young adults, who may be healthier on average, are eligible to take the medication. When investigating the effect of the medication on obesity, this is the proportion that should be used as the null hypothesis. If we observe an obesity rate on the medication of 40%, with a p-value less than the significance level of 0.05, this is evidence that the medication increases the risk of obesity (with an RR, in this scenario, of 1.6). As such, it is important to carefully choose the null hypothesis to make relevant statistical predictions.

With RR, a result of 1 signifies that both groups have the same amount of risk, while results not equal to 1 indicate that one group bore more risk than another, a risk that is assumed to be due to the intervention examined by the study (formally, the assumption of causal direction).

To illustrate, we look at the results of a 2009 study published in the Journal of Stroke and Cerebrovascular Diseases. The study reports that patients with a prolonged electrocardiographic QTc interval were more likely to die within 90 days compared with patients without a prolonged interval (relative risk [RR]=2.5; 95% confidence interval [CI] 1.5-4.1) [9]. Having a confidence interval between 1.5 and 4.1 for the risk ratio indicates that patients with a prolonged QTc interval were 1.5-4.1 times more likely to die in 90 days than those without a prolonged QTc interval.

A second example- in a landmark paper demonstrating that the blood pressure curve in acute ischemic stroke is U-shaped rather than J-shaped [10], the investigators found that the RR increased nearly two-fold in patients with mean arterial blood pressure (MAP) >140 mmHg or <100 mmHg (RR=1.8, 95% CI 1.1-2.9, p=0.027). Having a CI of 1.1-2.9 for the RR means that patients with a MAP outside the range of 100-140 mmHg were 1.1-2.9 times more likely to die than those who had initial MAP within this range.

For another example, a 2018 study on Australian naval recruits found that those with prefabricated orthoses (a type of foot support) had a 20.3% risk of suffering at least one adverse effect, while those without had a risk of 12.4% [11]. A risk ratio here is given by 0.203/0.124, or 1.63, suggesting that recruits with foot orthoses bore 1.63 times the risk of having some adverse consequence (e.g. foot blister, pain, etc.) than those without. However, the same study reports a 95% confidence interval for the risk ratio of 0.96 to 2.76, with a p-value of 0.068. Looking at the confidence interval, the 95% reported range (the commonly accepted standard) includes values under 1, 1, and values above 1. Remembering that all values are equally likely to be the population statistic, at 95% confidence, there is no way to exclude the possibility that foot orthoses have no effect, have a significant benefit, or have a significant detriment. Additionally, the p-value is greater than the standard of 0.05, therefore this data does not provide significant evidence of foot orthoses having any consistent effect on adverse events such as blisters and pain. As stated previously, this is no coincidence - if they are calculated using the same or similar methods and the p-value is two-tailed, confidence intervals and p-values will report the same results.

When utilized correctly, risk ratios are a powerful statistic that allow an estimation in a population of the change in risk one population bears over another. They are quite easy to understand (the value is how many times the risk one group bears over another), and with the assumption of causal direction, quickly show whether an intervention (or other tested variable) has an effect on outcomes.

However, there are limitations. Firstly, RRs cannot be applied in all cases. Because risk in a sample is an estimate of risk in a population, the sample must be reasonably representative of the population. As such, case-control studies, by simple virtue of the fact that ratios of outcomes are controlled, cannot have a risk ratio reported. Secondly, as with all the statistics discussed herein, RR is a relative measure, providing information about the risk in one group relative to another. The problem here is that a study where two groups had a risk of 0.2% and 0.1% bears the same RR, 2, as one where two groups had a risk of 90% and 45%. Though in both cases it is true that those with the intervention were at twice the risk, this equates to only 0.1% more risk in one case while 45% more risk in another case. Thus, reporting only the RR exaggerates the effect in the first instance, while potentially even minimizing the effect (or at least decontextualizing it) in the second instance.

Odds ratios

**Figure 1:**
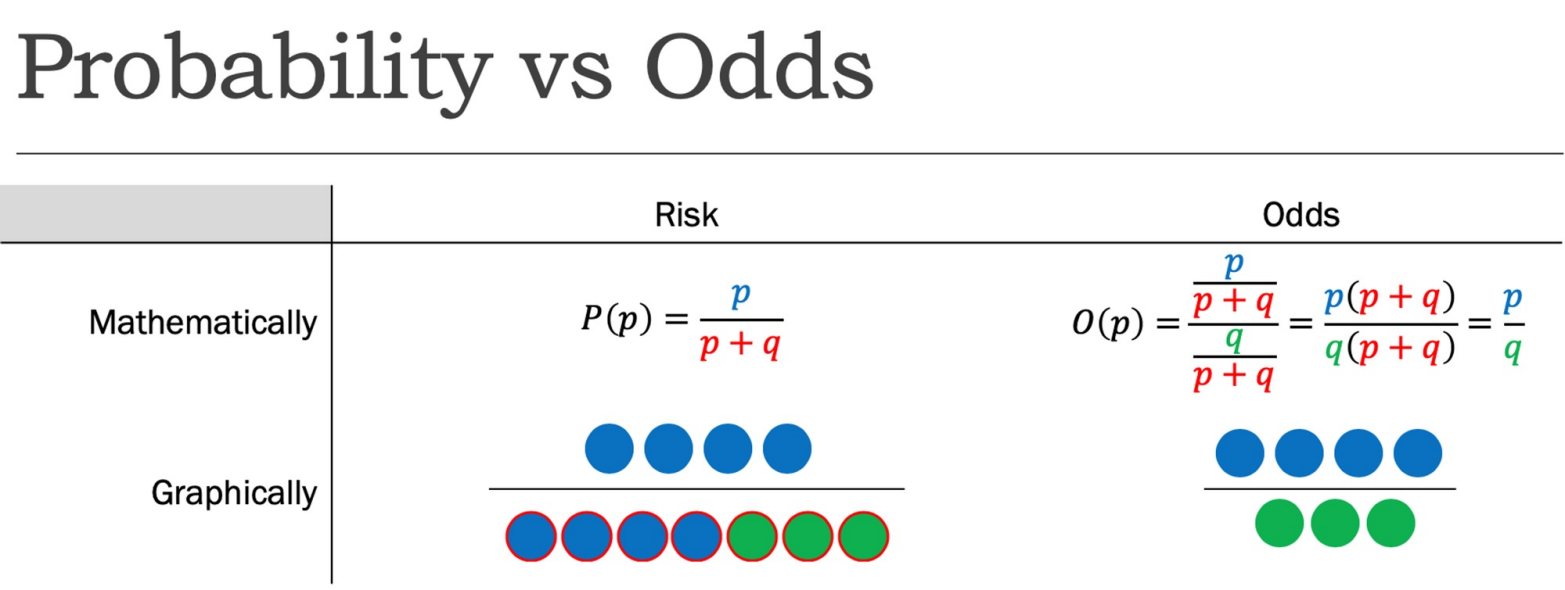
Probability (P) vs. Odds (O) where p=probability of success and q=probability of failure

While risk reports the number of events of interest in relation to the total number of trials, odds report the number of events of interest in relation to the number of events not of interest. Stated differently, it reports the number of events to nonevents. While the risk, as determined previously, of flipping a coin to be heads is 1:2 or 50%, the odds of flipping a coin to be heads is 1:1, as there is one desired outcome (event), and one undesired outcome (nonevent) (Figure 1). 

Just as with RR, where the ratio of two risks was taken for two separate groups, a ratio of two odds can be taken for two separate groups to produce an odds ratio (OR). Instead of reporting how many times the risk one group bears relative to the other, it reports how many times the odds one group bears to the other.

For most, this is a more difficult statistic to understand. Risk is often a more intuitive concept than odds, and thus understanding relative risks is often preferred to understanding relative odds. However, OR does not suffer from the same causal assumption limitations as RR, making it more widely applicable.

For instance, odds are a symmetric measure, meaning that while risk only examines outcomes given interventions, odds can also examine interventions given outcomes. Thus, a study can be constructed where, rather than choosing trial groups and measuring outcomes, outcomes can be chosen, and other factors can be analyzed. The following is an example of a case-control study, a situation where RR cannot be used but OR can.

A 2019 case-control study proves a good example. Seeking to find potential correlation between a hepatitis A virus (HAV) infection prominent in Canada and some causing factor, a study was constructed based on the outcome (in other words, individuals were categorized based on their HAV status, as the “intervention”, or causal event, was unknown). The study looked at those with HAV and those without and what foods they had eaten prior to HAV infection [12]. From this, multiple odds ratios were constructed comparing a specific food item to HAV status. For example, the data found that among those subjects who had exposure to shrimp/prawns, eight were positive for HAV while seven were not, while for those without exposure two were positive for HAV while 29 were not. An odds ratio is taken by (8:7)/(2:29) which equals approximately 16.6. The study data reported an OR of 15.75, with the small discrepancy likely originating from any pre-calculation adjustments for confounding variables that was not discussed in the paper. A p-value of 0.01 was reported, thus providing statistical evidence for this OR being significant.

This can be interpreted in two equal ways. Firstly, the odds of shrimp/prawn exposure for those with HAV are 15.75 times higher than for those without. Equivalently, the odds for HAV-posiitve versus HAV-negative is 15.75 times higher for those exposed to shrimps/prawns than for those not exposed.

Overall, OR provides a measure of the strength of association between two variables on a scale of 1 being no association, above 1 being a positive association, and below 1 being a negative association. While the previous two interpretations are correct, they are not as directly understandable as an RR would have been, had it been possible to determine one. An alternative interpretation is that there is a strong positive correlation between shrimp/prawn exposure and HAV.

Because of this, in some specific cases, it is appropriate to approximate RR with OR. In such cases, the rare disease assumption must hold. That is, a disease must be exceedingly rare within a population. Under this case, the risk of the disease within the population (p/(p+q)) approaches the odds of the disease within the population (p/q) as p becomes insignificantly small relative to q. Thus, the RR and OR converge as the population gets larger. However, if this assumption fails, the difference becomes increasingly exaggerated. Mathematically, in p+q trials, decreasing p increases q to maintain the same total trials. With risk, only the numerator changes, whereas with odds both the numerator and denominator change in opposite directions. As a result, for cases where the RR and OR are both below 1, the OR will underestimate the RR, while for cases where both are above 1, the OR will overestimate the RR.

Misreporting of the OR as the RR, then, can often exaggerate data. It is important to remember that OR is a relative measure just as RR, and thus sometimes a large OR can correspond with a small difference between odds.

For the most faithful reporting, then, OR should not be presented as an RR, and should only be presented as an approximation of RR if the rare disease assumption can reasonably hold. If possible, a RR should always be reported.

Hazard ratios

Both RR and OR concern interventions and outcomes, thus reporting across an entire study period. However, a similar but distinct measure, the hazard ratio (HR), concerns rates of change (Table 1).

**Table 1:** Relative risk (RR) vs. Odds Ratio (OR) vs. Hazard Ratio (HR)

	RR	OR	HR
Goal	Determine relationship in risk status based on some variable.	Determine association between two variables.	Determine how one group changes relative to another.
Use	Tells us how an intervention changes risks.	Tells us if there is an association between an intervention and risk; estimates how this association applies.	Tells us how an intervention changes the rate of experiencing an event.
Limitations	Only applicable if the study design is representative of the population. Cannot use on case-control studies.	Can generally be applied everywhere, but not always a useful statistic itself. Exaggerates risks.	To typically be useful, the rate of change within two groups should be relatively consistent.
Timeline	Static – does not consider rates. Summarizes an overall study.	Static – does not consider rates. Summarizes an overall study.	Based on rates. Provides information about the way a study progresses over time.

HRs are in tandem with survivorship curves, which show the temporal progression of some event within a group, whether that event is death, or contracting a disease. In a survivorship curve, the vertical axis corresponds to the event of interest and the horizontal axis corresponds to time. The hazard of the event is then equivalent to the slope of the graph, or the events per time.

A hazard ratio is simply a comparison of two hazards. It can show how quickly two survivorship curves diverge through comparison of the slopes of the curves. An HR of 1 indicates no divergence - within both curves, the likelihood of the event was equally likely at any given time. An HR not equal to 1 indicates that two events are not occurring at an equal rate, and the risk of an individual in one group is different than the risk of an individual in another at any given time interval.

An important assumption that HRs make is the proportional rates assumption. To report a singular hazard ratio, it must be assumed that the two hazard rates are constant. If the slope of the graph is to change, the ratio will likewise change over time, and thus will not apply as a comparison of likelihood at any given time.

Consider the trial of a novel chemotherapeutic agent seeking to extend life expectancy of patients with a specific cancer. In both the intervention and the control group, 25% had died by week 40. Since both groups decreased from 100% survival to 75% survival over the 40-week period, the hazard rates would be equal and thus the hazard rate equal to 1. This suggests that an individual receiving the drug is just as likely to die as one not receiving the drug at any time.

However, it is possible that in the intervention group, all 25% died between weeks six to 10, while for the control group, all 25% died within weeks one to six. In this case, comparing medians would display a higher life expectancy for those on the drug despite the HR not showing any difference. In this case, the proportional hazards assumption fails, as the hazard rates change (quite dramatically) over time. In cases such as this, HR is not applicable.

Because it is sometimes difficult to determine whether the proportional hazards assumption reasonably applies, and because taking an HR strips the original measurement (hazard rates) of the time unit, it is common practice to report HR in conjunction with median times.

In a study evaluating the prognostic performance of The Rapid Emergency Medicine Score (REMS) and the Worthing Physiological Scoring system (WPSS), the investigators found that the risk of 30-day mortality was increased by 30% for each additional REMS unit (HR: 1.28; 95% confidence interval (CI): 1.23-1.34) and by 60% for each additional WPSS unit (HR: 1.6; 95% CI: 1.5-1.7). In this case, the death rate did not change, but rather the scoring system to predict it did, so the HR can be used. Having a confidence interval between 1.5 and 1.7 for the WPSS hazards ratio indicates that the mortality curve for those with a higher WPS declines at a faster rate (about 1.5-1.7 times). Since the low end of the interval is still above 1, we are confident that the true hazard of death within 30 days is higher for the group with higher WPS [13].

In a 2018 study on binge drinking amongst individuals with certain risk factors, a survival curve was constructed plotting the rate of achieving binge drinking for controls, those with a family history, male sex, those with high impulsivity, and those with a higher response to alcohol. For men and those with a family history, statistically significant evidence for a higher rate of achieving binge drinking was reported (an HR of 1.74 for men and 1.04 for those with a family history) [14]. However, for those with high impulsivity, though the HR was 1.17, the 95% confidence interval ranged from 1.00 to 1.37. Thus, to a 95% confidence level, it is impossible to rule out that the HR was 1.00.

Because of the exaggeration present, it is important to avoid representing ORs as RRs, and similarly, it is important to recognize that a reported OR rarely provides a good approximation of relative risks but rather simply provides a measure of correlation.

Because of its ability to make firm conclusions and understandability, RR should be reported if possible, however in the cases where its causality assumption is violated (such as case-control studies and logistic regression), OR can be used.

HRs are used with survival curves and assume that hazard rates are equal over time. While useful to compare two rates, they should be reported with median times to justify the proportional hazards assumption.

**Figure 2:**
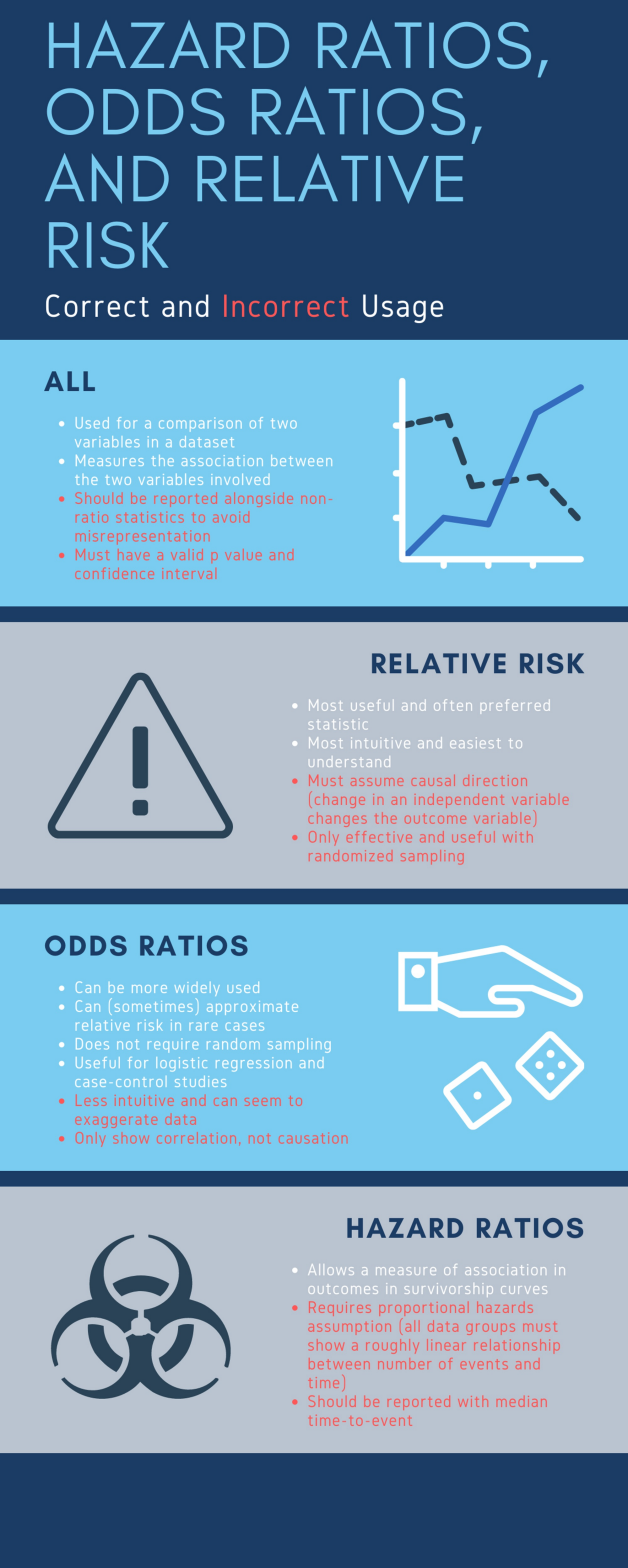
Correct and incorrect usage of Hazard Ratios (HR), Odds Ratios (OR) and Relative Risk (RR)

Finally, regardless of the value of the HR/RR/OR statistic, an interpretation should only be made after determining whether the result provides statistically significant evidence towards a conclusion (as determined by the p-value or confidence interval). Remembering these principles and the framework of HR/RR/OR minimizes misrepresentation and prevents one from drawing incorrect conclusions from the results of a published study concerning various samples. Figure 2 summarizes correct and incorrect usage of these various risk ratios.

## Conclusions

Medical literature is full of statistical measures designed to help clinicians make inferences about a particular intervention or association between variables or the effect of an intervention over time. Thus understanding the meaning of each of these measures is paramount for making patient care decisions.
